# Prevalence and characteristics of alcohol consumption and risk of type 2 diabetes mellitus in rural China

**DOI:** 10.1186/s12889-021-11681-0

**Published:** 2021-09-09

**Authors:** Xueyan Wu, Xiaotian Liu, Wei Liao, Ning Kang, Xiaokang Dong, Tanko Abdulai, Zhihan Zhai, Chongjian Wang, Xiaoqiong Wang, Yuqian Li

**Affiliations:** 1 Department of Epidemiology and Biostatistics, College of Public Health, Zhengzhou University, 100 Kexue Avenue, Zhengzhou, 450001 Henan P. R. China; 2 Department of Preventive Medicine, Henan University of Chinese Medicine, Zhengzhou, Henan P. R. China; 3 Department of Economics, School of Business, Zhengzhou University, Zhengzhou, Henan P. R. China; 4 Department of Clinical Pharmacology, School of Pharmaceutical Science, Zhengzhou University, Zhengzhou, Henan P. R. China

**Keywords:** Alcohol, Abstinence, WHO drinking risk levels, Type 2 diabetes mellitus, Rural population

## Abstract

**Background:**

The study aimed to characterize the prevalence of alcohol consumption and further investigate the relationship between alcohol consumption and type 2 diabetes mellitus (T2DM).

**Methods:**

We studied 39,259 participants aged 18 to 79 years of the Henan Rural Cohort study. The associations between alcohol consumption and T2DM were examined using the logistic regression models and restricted cubic spline.

**Results:**

For men, alcohol abstinence was associated with an increased risk of T2DM (1.491(1.265, 1.758)), whereas current drinkers were not associated with T2DM (1.03(0.91, 1.15)). Further analysis of alcohol drinkers revealed that only high-risk drinkers of WHO drinking risk levels increased the risk of T2DM (1.289(1.061,1.566)) compared to never drinkers. The risk of T2DM increased as the age of starting to consume alcohol decreased and as the number of years of consuming alcohol and the alcohol intake increased only in men. We further found that the risk of T2DM decreased as the number of years of abstinence increases and no association between alcohol abstinence and T2DM was found after more than 10 years of abstinence among men.

**Conclusions:**

Our results suggested that reducing the amount of alcohol consumed and adhering to abstinence from alcohol consumption are beneficial in reducing the risk of T2DM.

**Trial registration:**

The Henan Rural Cohort Study has been registered at Chinese Clinical Trial Register (Registration number: ChiCTR-OOC-15006699). Date of registration: 2015-07-06. http://www.chictr.org.cn/showproj.aspx?proj=11375

**Supplementary Information:**

The online version contains supplementary material available at 10.1186/s12889-021-11681-0.

## Background

Alcohol consumption and health have a complex connection. Alcohol consumption is one of the leading risk factors for death and disease worldwide, and studies link its consumption to 60 acute and chronic diseases [1–4]. Since 2000, the percentage of people who drink alcohol globally has fallen from 47.6 to 43.0%, a decline of nearly 5% [4]. Nevertheless, the percentage of current drinkers is rising, in the Western Pacific region dominated by China [5]. Per capita alcohol consumption in China rose from 4.1 l in 2005 to 7.1 l in 2010, which was higher than the world average [4]. Compared to other countries, China has a different demographic profile and a specific “drinking culture”. Alcohol consumption is very common during important festivals, business occasions, ceremonies, and special events in China. The Survey on the Health and Nutrition Status of the Chinese Population showed that the alcohol consumption rate in China in the past year was about 34% [6]. Moreover, alcohol consumption is a normal part of the daily diet, especially in rural areas of China.

China has a large burden of diabetes in recent years [7]. Diabetes is a major public health issue in China, especially type 2 diabetes mellitus (T2DM). China has a large health burden of diabetes: in 2013, a quarter of diabetic patients worldwide were in China, where 11.6% of adults had diabetes and 50.1% had prediabetes [8]. Alcohol consumption was one of the risk factors of T2DM. However, the existing literature on the relationship between alcohol consumption and T2DM is inconsistent and controversial. The majority of the studies revealed that moderate alcohol consumption with a decrease in the risk of diabetes, whereas high alcohol intake was associated with a higher risk [9–15]. There are still a few studies that show that alcohol consumption increases the incidence of T2DM, regardless of the amount of alcohol consumed [16–18]. Nevertheless, the definitions of moderate and high alcohol intake have varied across studies. World Health Organization (WHO) risk drinking levels is associated with health beneficial effects [19, 20]. In a US national survey with follow-up studies, a reduction in WHO high-risk drinking levels was found to be associated with a reduced risk of cardiovascular disease among very-high-risk and high-risk drinkers, further demonstrating that reducing high levels of alcohol consumption has important benefits in multiple clinical areas [21]. However, no studies have been found on the relationship between WHO risk drinking levels of alcohol consumption and diabetes.

In addition, few studies have been conducted on resource-constrained areas. As a result, we conducted a large population-based study to characterize the prevalence of alcohol consumption and to further investigate the relationship between different lengths of abstinence and different WHO drinking risk levels of alcohol consumption and T2DM in rural areas of China.

## Methods

### Study design and participants

This study population is from the Henan Rural Cohort Study. From July 2015 to September 2017, the cohort was conducted in Yuzhou county, Suiping county, Xinxiang county, Kaifeng county, and Yima county of Henan Province in China. A multi-stage, stratified cluster sampling method was used to select the sample. Rural areas with signed informed consent forms were selected as the study sample. The details of this cohort have been introduced elsewhere [22]. Finally, 39,259 subjects were included in this study. Written informed consent was obtained from each participant prior to data collection.

The Henan Rural Cohort Study was approved by the Zhengzhou University Life Science Ethics Committee (Code: [2015] MEC (S128)), and was conducted according to the 1975 Declaration of Helsinki.

### Data collection

Detailed information about sociodemographic characteristics (age, gender, education level, marital status, and per capita monthly income), lifestyle factors (smoking, alcohol consumption, and physical activity), and personal history of chronic diseases was collected through face-to-face interviews with standardized questionnaires [23]. The physical activity was assessed using International Physical Activity Questionnaire (IPAQ 2001) [24]. Data on diet intake via a validated Food Frequency Questionnaire (FFQ) [25]. The FFQ based on the Dietary Guidelines for Chinese Residents and the eating habits.

The height and weight of participants were measured twice, and the average readings were computed to analyze. Body mass index (BMI, kg/m^2^) was calculated as weight (kg) divided the square of height (m). Blood pressure was measured three times by electronic sphygmomanometer in the right arm in a sitting position after at least 5 min rest. There were 30s intervals between the three measurements. Venous blood samples were collected from subjects after an overnight fast of at least 8 h and stored in − 80 °C cryogenic refrigerator before analysis. The fasting blood glucose (FBG) was analyzed via glucose oxidative method (GOD-PAP) by ROCHE Cobas C501 automatic biochemical analyzer. Total cholesterol was measured by Roche Cobas C501 automatic biochemical analyzer. The details of the equipment for anthropometric and clinical examinations have been introduced elsewhere [22]. T2DM Patients were defined as those who had an FPG ≥ 7.0 mmol/L, or diagnosed T2DM by physicians and using anti-glycemic drugs in the last 2 weeks [26].

### Alcohol consumption estimation

Alcohol consumption was collected through a face-to-face interview questionnaire, which included screening questions to distinguish current drinkers, former drinkers, and lifetime abstainers, and parallel question groups on the types of drinking (beer, liquor, red wine, and rice wine). Participants were asked whether they had ever consumed alcoholic drinks, and if they had, they were asked whether they were former-drinkers or current-drinkers. For current-drinkers, they were additionally asked to fill out a questionnaire asking about the amount and frequency of alcohol consumption. Participants were asked about the frequency (daily, weekly, monthly, yearly, or never) and amount of their consumption of every kind of wine in the past 12 months. Total daily alcohol consumption was calculated using the average frequency, amount per occasion, and alcohol content. Consistent with the Chinese Dietary Guidelines summary [27], we calculated WHO drinking risk levels [28] based on participant’s reports of the number of standard drinks consumed, which were transformed into grams of pure alcohol. The 4 risk levels are defined as following: high-risk drinkers (> 60 g/d for men, > 40 g/d for women); medium-risk drinkers (> 25 to 60 g/d for men, > 15 to 40 g/d for women), low-risk (> 0 to 25 g/d for men,> 0 to 15 g/d for women), and non-drinkers [28].

### Statistical analysis

Continuous variables were expressed as mean and standard deviation, whereas categorical variables were expressed as percentages. All analyses were performed using the student t-test, Pearson’s correlation coefficient, or one-way analysis of variance test. To further visually observe association between the changes of age of starting to consume alcohol and alcohol intake and T2DM, multivariable restricted cubic regression splines with three knots placed at the fifth, 50th and 95th was performed by SAS version 9.1 (RCS_Reg v1.0.sas) and R version 3.6.3 (ggplot2). The multivariable logistic regression model was conducted to investigate the relationship between drinking status, duration of abstinence, WHO drinking risk levels and risk of T2DM, and three models were established: Model 1 was unadjusted; Model 2 was adjusted for age, marital status, average monthly individual income, education level, smoking, more vegetables and fruits intake, high fat diet for the variables in the Model 1 plus dyslipidemia, hypertension, and family histories of hypertension and T2DM; Model 3 was adjusted for the variables in the Model 2 plus more vegetables and fruits intake, high- fat diet, physical activity, and family histories of T2DM. *P* values < 0.05 were considered statistically significant. All statistical analyses were performed using SPSS software V.26.0, SAS version 9.1 (RCS_Reg v1.0.sas), and R version 3.6.3.

## Results

### Characteristics of the studied population

**Fig. 1:**
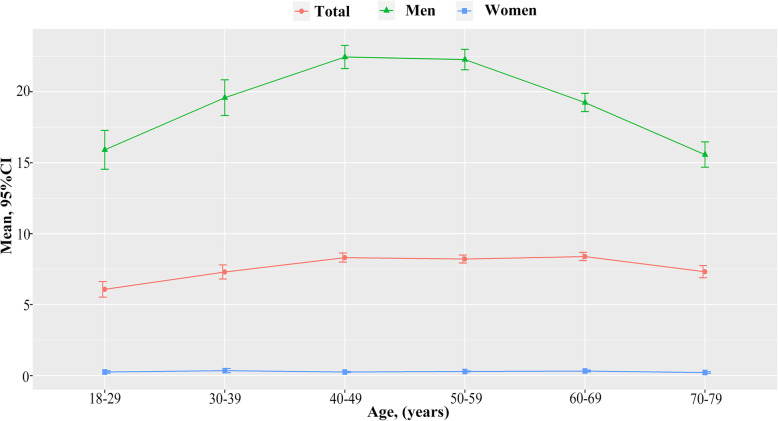
Mean (95%CI) of alcohol

Of all 39,259 participants, 23,769 (60.54%) were women and the mean age was 55.60 ± 12.19 years. 3708 (1411 in men and 2297) cases of T2DM were identified. The prevalence of never drinkers, former drinkers, and current drinkers was 77.30% (46.85% in men and 97.14% in women), 4.67% (11.41% in men and 0.27% in women), and 18.03% (41.74% in men and 2.59% in women), respectively. Lower age, higher education levels, income, SBP and DBP, higher proportion of current smokers and obesity were observed among those who were current drinkers compared to non-current drinkers. Similar results were observed in men, while higher proportion of overweight, lower TC and FPG were observed among women who were current drinkers compared to non-current drinkers (Table 1 and Table S1). The WHO risk drinking levels of high-risk drinkers, medium-risk drinkers and low-risk drinkers were 3.90% (9.63% in men and 0.17% in women), 4.71% (11.38% in men and 0.36% in women) and 13.87% (31.69% in men and 2.26% in women), respectively (Table S2). A summary of participants’ age of starting to drink, duration of drinking and alcohol intake by sex is show in Table S3. The mean alcohol intake was 19.94 g/d in men (0.29 g/d in women, ranging from > 0 to 246.57 g/d), ranging from > 0 to 532.60 g/d. The mean age of starting to drink was 22.45 years in men and 33.37 years in women. The mean duration of drinking was 31.61 years in men and 23.37 years in women. Figure 1 displays the sex-specific distributions of alcohol consumption according to age. There were statistically significant U-shaped associations between the mean levels of alcohol consumption and age groups in men, with the highest mean values among participants with the age of 40 to 49. Women’s alcohol intake was very low and did not change much with age. Fig. S1 and Table S4 show the distribution of age of starting to drink and initiation of sobriety. The percentage of people who started drinking alcohol before the age of 18 reached 25.96% (26.99% in men and 11.41% in women) and the lowest age of starting to drink was 7. The most prevalent of age group was 18 to 29 both in men (58.46%) and women (33.16%). The proportion of women who start drinking after the age of 30 was higher than that of men. In addition, most men begin to stop drinking at age 50 to 59, while most women begin abstaining at age 40 to 49. Further exploring the reasons for quitting (Fig. S2), we found that most people quit drinking for health reasons such as disease. Furthermore, alcoholic beverages mainly consisted of liquor and beer. Beer intake decreased with age, while liquor consumption increased (Fig. S3).

**Table 1:** Characteristics of the participants by drinking status

Characteristic, n (%)	Total (***n*** = 39,259)	Drinking status			***P***
		Never drinker	Former drinker	Current drinker	
**Total**	39,259 (100.00)	30,347 (77.30)	1832 (4.67)	7080 (18.03)	< 0.001
**Age (years, mean ± SD)**	55.60 ± 12.19	55.89 ± 12.17	60.61 ± 10.10	53.05 ± 12.20	< 0.001
**Sex**					< 0.001
Men	15,490 (39.46)	7257 (46.85)	1768 (11.41)	6465 (41.74)	
Women	23,769 (60.54)	23,090 (97.14)	64 (0.27)	615 (2.59)	
**Marital status**					< 0.001
Married/cohabiting	35,243 (89.77)	27,036 (76.71)	1656 (4.70)	6551 (18.59)	
Unmarried/divorced/widowed	4016 (10.23)	3311 (82.45)	176 (4.38)	529 (13.17)	
**Education levels**					< 0.001
Primary school or illiteracy	17,572 (44.76)	14,848 (84.50)	707 (4.02)	2017 (11.48)	
Junior high school	15,643 (39.85)	11,415 (72.97)	807 (5.16)	3421 (21.87)	
High school or above	6044 (15.40)	4084 (67.57)	318 (5.26)	1642 (27.17)	
**Income (RMB per month)**					< 0.001
< 500	14,014 (35.70)	11,136 (79.36)	753 (5.37)	2125 (15.16)	
500~	12,907 (32.88)	10,023 (77.66)	576 (4.46)	2308 (17.88)	
1000~	12,338 (31.43)	9188 (74.47)	503 (4.08)	2647 (21.45)	
**Smoking**					< 0.001
Never	28,580 (72.80)	26,235 (91.79)	273 (0.96)	2072 (7.25)	
Former	3192 (8.13)	1184 (37.09)	827 (25.91)	1180 (37.00)	
Current	7487 (19.07)	2928 (39.11)	732 (9.78)	3827 (51.12)	
**Physical activity**					< 0.001
Low	12,715 (32.39)	9646 (75.86)	754 (5.93)	2315 (18.21)	
Moderate	14,805 (37.71)	12,315 (83.18)	513 (3.47)	1977 (13.35)	
High	11,739 (29.90)	8386 (71.44)	565 (4.81)	2788 (23.75)	
**BMI**					< 0.001
Underweight	952 (2.43)	787 (82.67)	51 (5.36)	114 (11.97)	
Normal	15,749 (40.25)	12,209 (77.52)	808 (4.54)	2732 (18.58)	
Overweight	15,479 (39.56)	11,901 (76.88)	702 (38.57)	2876 (40.73)	
Obesity	6951 (17.76)	5353 (77.01)	259 (3.73)	1339 (19.26)	
**SBP (mmHg, mean ± SD)**	125.96 ± 19.99	125.74 ± 20.41	127.51 ± 19.44	126.49 ± 18.20	< 0.001
**DBP (mmHg, mean ± SD)**	77.70 ± 11.64	77.10 ± 11.44	78.07 ± 11.86	80.14 ± 12.12	< 0.001
**TC (mmol/L, mean ± SD)**	4.76 ± 0.99	4.78 ± 0.99	4.57 ± 0.97	4.70 ± 0.97	< 0.001
**FPG (mmol/L, mean ± SD)**	5.54 ± 1.51	5.54 ± 1.51	5.64 ± 1.63	5.52 ± 1.47	0.013
**T2DM (yes)**	3708 (1000))	2924 (78.86)	239 (6.45)	545 (14.70)	< 0.001

*Abbreviation*s: *SD* standard deviation, *RMB* Renminbi, *BMI* body mass index, *SBP* systolic blood pressure, *DBP* diastolic blood pressure, *TC* total cholesterol, *FPG* fasting plasma glucose, *T2DM* type 2 diabetes mellitus

### Associations between alcohol consumption and T2DM

The association between alcohol consumption and T2DM according sex is shown in Table 2. For men, after adjusting for the potential confounding factors, compared to non-drinkers, alcohol abstinence was associated with an increased risk of T2DM (1.49(1.27, 1.76)), while current drinkers were not associated with T2DM (1.00(0.88, 1.13)). Further analysis of alcohol drinkers revealed that only high-risk drinkers increased the risk of T2DM (1.289(1.061,1.566)) compared to never drinkers. In women, no association was found between alcohol abstinence and T2DM. however, alcohol consumption might reduce the risk of T2DM compared with never drinkers among rural women. In addition, compared to non-drinkers, the association between alcohol consumption and T2DM was observed only in low-risk drinkers for women. Multivariable restricted cubic regression splines were conducted with three knots placed at the fifth, 50th and 95th to visually explore the association between age of starting to consume alcohol, number of years of consuming alcohol and T2DM. The odds of T2DM increased with the increment of number of years of consuming alcohol (Fig. S4) and alcohol intake (Fig. S5) and with the decrement of the age of starting to consume alcohol (Fig. S6) in total population and men after adjusting potential confounders. In addition, we analyzed the relationship between drinking types and T2DM (Table S5). Of the many types of alcoholic beverages, only liquor might increase the risk of T2DM (1.002(1.001, 1.003)) among men. No association between type of alcoholic and T2DM was found in women.

**Table 2:** The association of drinking status with T2DM by sex

Drinking status	N (%)	Model 1	Model 2	Model 3
		OR (95%CI)	OR (95%CI)	OR (95%CI)
**Total**				
Never drinker	30,347 (77.30)	1 [Reference]	1 [Reference]	1 [Reference]
Former drinker	1832 (4.67)	1.41 (1.22, 1.62)*	1.45 (1.24, 1.70)*	1.42 (1.21, 1.66)*
Current drinker	7080 (18.03)	0.78 (0.71, 0.86)*	1.04 (0.92, 1.16)	1.03 (0.91, 1.15)
**WHO risk drinking levels**				
Low-risk drinkers	5447 (61.71)	0.84 (0.76, 0.94)*	1.06 (0.94, 1.19)	1.04 (0.92, 1.18)
Medium-risk drinkers	1848 (20.94)	0.96 (0.82, 1.13)	1.23 (1.03, 1.46)*	1.22 (1.02, 1.46)*
High-risk drinkers	1532 (17.36)	1.07 (0.91, 1.27)	1.35 (1.12, 1.62)*	1.32 (1.10, 1.60)*
**Men**				
Never drinker	7257 (46.85)	1 [Reference]	1 [Reference]	1 [Reference]
Former drinker	1768 (11.41)	1.49 (1.27, 1.74)*	1.52 (1.29, 1.79)*	1.49 (1.27, 1.76)*
Current drinker	6465 (41.74)	0.85 (0.76, 0.96)*	1.00 (0.88, 1.14)	1.00 (0.88, 1.13)
**WHO risk drinking levels**				
Low-risk drinkers	4909 (60.13)	0.92 (0.81, 1.05)	1.06 (0.93, 1.21)	1.06 (0.92, 1.21)
Medium-risk drinkers	1763 (21.59)	1.03 (0.86, 1.23)	1.19 (0.99, 1.43)	1.18 (0.98, 1.42)
High-risk drinkers	1492 (18.28)	1.13 (0.94, 1.36)	1.32 (1.09, 1.60)*	1.29 (1.06, 1.57)*
**Women**				
Never drinker	23,090 (97.14)	1 [Reference]	1 [Reference]	1 [Reference]
Former drinker	64 (0.27)	1.32 (0.63, 2.77)	1.35 (0.63, 2.88)	1.26 (0.59, 2.71)
Current drinker	615 (2.59)	0.51 (0.35, 0.72)	0.60 (0.42, 0.86)*	0.57 (0.40, 0.82)*
**WHO risk drinking levels**				
Low-risk drinkers	538 (81.15)	0.55 (0.38, 0.79)*	0.66 (0.45, 0.96)*	0.61 (0.42, 0.88)*
Medium-risk drinkers	85 (12.82)	0.70 (0.31, 1.61)	0.67 (0.29, 1.56)	0.75 (0.32, 1.75)
High-risk drinkers	40 (6.03)	1.03 (0.35, 2.88)	1.04 (0.36, 2.98)	1.05 (0.37, 3.02)

*OR* odds ratios, *CI* confidence intervals

*, *P* < 0.05

WHO risk drinking levels, low-risk drinkers (> 0 to 25 g/d for men,> 0 to 15 g/d for women), medium-risk drinkers (> 25 to 60 g/d for men, > 15 to 40 g/d for women), high-risk drinkers (> 60 g/d for men, > 40 g/d for women)

Model 1: unadjusted; Model 2: adjusted for age, gender, education level, marital status, and smoking; Model 3: adjusted for model 2 plus family histories of T2DM, more vegetables and fruits intake, high fat diet, physical activity

### Associations between number of years of abstinence from alcohol and T2DM

Table 3 shows the association of abstinence duration with T2DM by sex. We further found that the risk of T2DM decreased as the number of years of abstinence increases and no association between alcohol abstinence and T2DM was found after more than 10 years of abstinence in men. No association between abstinence duration and T2DM was found among women.

**Table 3:** The association of abstinence duration with T2DM by sex

Abstinence duration	N (%)	Model 1	Model 2	Model 3
		OR (95%CI)	OR (95%CI)	OR (95%CI)
**Total**				
Never drinker	30,347 (77.30)	1 [Reference]	1 [Reference]	1 [Reference]
≤ 1^**a**^	372 (0.95)	1.59 (1.19, 2.13)*	1.84 (1.36, 2.49)*	1.74 (1.28, 2.37)*
2-5^**a**^	550 (1.40)	1.50 (1.18, 1.92)*	1.66 (1.29, 2.15)*	1.66 (1.28, 2.16)*
6-10^**a**^	357 (0.91)	1.45 (1.07, 0.98)	1.52 (1.10, 2.09*)	1.51 (1.10, 2.08)*
11-15^**a**^	204 (0.52)	1.38 (0.91, 2.08)	1.43 (0.94, 2.19)	1.43 (0.94, 2. 20)
≥ 16^**a**^	337 (0.86)	1.02 (0.71, 1.47)	0.90 (0.62, 1.30)	0.87 (0.60, 1.26)
**Men**				
Never drinker	7257 (46.85)	1 [Reference]	1 [Reference]	1 [Reference]
≤ 1^**a**^	358 (2.31)	1.68 (1.24, 2.27)*	1.80 (1.32, 2.44)*	1.68 (1.23, 2.30)*
2-5^**a**^	526 (3.40)	1.61 (1.25, 2.09)*	1.66 (1.28, 2.15)*	1.66 (1.28, 2.17)*
6-10^**a**^	345 (2.23)	1.48 (1.07, 2.04)*	1.48 (1.07, 2.06)*	1.49 (1.07, 2.08)*
11-15^**a**^	199 (1.28)	1.49 (0.98, 2.27)	1.50 (0.98, 2.28)	1.52 (0.99, 2. 33)
≥ 16^**a**^	330 (2.13)	1.07 (0.73, 1.55)	0.99 (0.68, 1.45)	0.96 (0.66, 1.41)
**Women**				
Never drinker	23,090 (97.14)	1 [Reference]	1 [Reference]	1 [Reference]
≤ 1^a^	14 (0.06)	1.54 (0.34, 6.87)	1.53 (0.33, 7.21)	1.43 (0.30, 6.76)
2-5^a^	24 (0.10)	0.84 (0.20, 3.57)	1.12 (0.26, 4.90)	1.07 (0.24, 4.69)
6-10^a^	12 (0.05)	3.07 (0.83, 11.35)	2.68 (0.71, 10.07)	2.30 (0.60, 8.78)
11-15^a^	5 (0.02)	*NA*	*NA*	*NA*
≥ 16^a^	7 (0.03)	1.54 (0.19, 12.76)	1.02 (0.12, 8.62)	0.99 (0.12, 8.31)

*OR* odds ratios, *CI* confidence intervals

^a^Abstinence duration, year. *, *P* < 0.05

Model: unadjusted; Model 2: adjusted for age, gender, education level, marital status, and smoking; Model 3: adjusted for model 2 plus family histories of T2DM, more vegetables and fruits intake, high fat diet, physical activity

## Discussion

In the present study, the prevalence of alcohol consumption was much higher in men (41.74%) than in women (2.59%). There was a statistically significant U-shaped association between mean alcohol consumption level and age group in men, with the highest in the 40–49 years group. Higher rates of initiation of alcohol consumption before age 18 (26.99% in men and 11.41% in women). Most people start alcohol consumption at the age of 18 to 29 (58.46% in men and 33.16% in women). In addition, the vast majority of people quit drinking for health reasons such as disease (88.37% in men and 62.30 in women). For men, abstinence from alcohol was associated with an increased risk of T2DM, whereas current drinkers were not associated with T2DM. Further analysis of alcohol drinkers revealed that only high-risk drinkers of WHO drinking risk levels increased the risk of T2DM. The risk of T2DM increased as the age of starting to consume alcohol decreased and as the number of years of consuming alcohol and the alcohol intake increased only in men. The association between alcohol consumption and T2DM was only observed in women who were low-risk drinkers. In addition, only liquor was associated with a higher risk of T2DM in men, while no association between alcohol type and T2DM was found in women. We further found that the risk of T2DM decreased as the number of years of abstinence increases and no association between alcohol abstinence and T2DM was found after more than 10 years of abstinence among men.

There are many studies that confirm higher rates of alcohol consumption in rural areas than in urban areas [29, 30]. Liqueur consumption was more common in rural China. This might be related to the emergence of large amounts of homemade alcohol in rural China [31], which might further increase the consumption of alcohol among rural men [32]. This also suggests that more attention is given to the problem of alcohol consumption in rural areas and that active control measures are needed to intervene in alcohol consumption. Our study found a significant U-shaped association between mean levels of alcohol consumption and age group in men, with the highest mean values for participants aged 40 to 49 years. In contrast, one study showed that drinking rates increased with age, with higher rates in the older age groups [31]. This discrepancy might be the result of a declining workforce among older adults without retirement pay to the extent that they can hardly afford daily alcohol expenditures. The trend of liquor consumption with age was the opposite of beer, with more rural Chinese men preferring to consume liquor as they get older. In China, young people preferred to drink beer, and older people might use liqueurs in social gatherings to enhance the atmosphere or add emotion [30]. In addition, we found that 25.96% started drinking before the age of 18 and the lowest age of starting to drink was 3. Alcohol use is prevalent among adolescents in China [33]. This might be because rural populations are not fully aware of the dangers of alcohol consumption. More public measures should be established to address the problem of underage drinking.

Our study found that reducing the amount of alcohol consumed or adhering to abstinence from alcohol was advantageous in reducing the risk of T2DM. Previous studies have found that moderate alcohol use was associated with a lower incidence of diabetes [12, 34–36]. There was evidence that moderate consumption of alcohol increases insulin sensitivity [37, 38]. Moderate alcohol consumption once a day significantly reduces fasting blood glucose levels [39]. Increasing alcohol consumption was significantly associated with lower glycated hemoglobin concentrations [40]. A study has indicated that light and moderate alcohol consumption weakens the link between obesity and glycemic status [41], which of one possible explanation is that alcohol suppresses obesity-induced insulin resistance. An experimental study in mice fed a high-fat diet showed that alcohol increased insulin sensitivity by upregulating anti-insulin sensitivity genes and that obesity-related alterations in insulin sensitivity were combined with alterations in insulin sensitivity genes [42]. Moderate alcohol consumption may inhibit obesity-induced insulin resistance by increasing lipocalin, growth hormone-releasing peptide, and anti-inflammatory molecules [42, 43]. Nevertheless, several other studies have suggested that moderate alcohol consumption was not associated with a reduced risk of developing T2DM [44, 45]. The mechanisms underlying the beneficial effects of moderate alcohol consumption on diabetes still require further in-depth study. With ongoing research, a recent meta-analysis revealed that the reduced risk for moderate drinkers may be limited to women and non-Asian populations [15], which was similar to our findings that low-risk drinking levels might reduce the risk of T2DM only in women, and our results added to the evidence of rural populations in China. In addition, we conducted the first study on the relationship between alcohol consumption and T2DM in rural Chinese. And we found that high-risk drinking levels might increase the risk of T2DM, however, we did not find any association between low-risk drinking level or medium-risk drinking level and T2DM. The study of the JPHC study cohort I found similar results that moderate to high alcohol consumption was positively associated with the incidence of diabetes in Japanese [46]. Our study further supports that reducing alcohol consumption [19, 20] are beneficial in reducing the risk of T2DM in high-risk drinkers, although no association was found between low-risk drinking level or medium-risk drinking level and T2DM. Our research supports the beneficial effects of abstinence and adherence to abstinence might counteract the harmful effects of alcohol consumption. Our further study found that alcohol abstinence increased the risk of T2DM, while current drinkers were not associated with T2DM. This was consistent with the findings of a study of African American women [47]. Moreover, the risk of T2DM decreased as the number of years of abstinence increases and no association between alcohol abstinence and T2DM was found after more than 10 years of abstinence. The reason for this result might be that our research showed that the vast majority of reasons for abstaining from drinking were due to health reasons such as illness [48]. And current drinkers might be in relatively good health. In addition to this we also found that the risk of T2DM increased as the age of starting to consume alcohol decreased and as the number of years of consuming alcohol and the alcohol intake increased only in men. Our results further demonstrated the harmful effects of alcohol. The later you start drinking, the shorter you drink, and the smaller the amount of alcohol you drink, the more beneficial it is to your health.

The limitations of our study should be considered. First, our findings were from a cross-sectional study and therefore do not accurately describe causality. Secondly, the use of self-report questionnaires might lead to recall bias in the collection of data. Thirdly, some participants might have given subjectively evasive answers to questions about alcohol consumption. Alcohol consumption might be underestimated. In addition, the sample size of women drinking participants was relatively small. The impact of alcohol consumption on the health of the women population needs to be further explored.

## Conclusion

The risk of T2DM increased as the age of starting to consume alcohol decreased and as the number of years of consuming alcohol and the alcohol intake increased only in men. After grouping abstainers by the duration of abstinence and grouping drinkers by the amount of alcohol they consumed, we further found that the risk of T2DM decreased as the number of years of abstinence increases and no association between alcohol abstinence and T2DM was found after more than 10 years of abstinence among men. Our findings indicated that reducing the amount of alcohol consumed and adhering to abstinence from alcohol consumption is beneficial in reducing the risk of T2DM.

## Supplementary Information

**Additional file 1.**

## Data Availability

The datasets used and/or analyzed during the current study available from the corresponding author on reasonable request.

## Abbreviations

T2DM: Type 2 diabetes mellitus
WHO: World Health Organization
MI: Body mass index
FBG: Fasting blood glucose
