# Supplementary material for: Prevalence and characteristics of alcohol consumption and risk of type 2 diabetes mellitus in rural China
Source: BMC Public Health. 2021 Sep 9;21:1644. doi: 10.1186/s12889-021-11681-0 (PMC8428056; doi:10.1186/s12889-021-11681-0)
Supplement: Supplementary file 1 — Additional file 1. [file 12889_2021_11681_MOESM1_ESM.docx]

**Supplementary Table 1. Characteristics of the participants according drinking status by sex.**

| **Characteristic, n (%)** | **Men** | | | |  | **Women** | | | |
| --- | --- | --- | --- | --- | --- | --- | --- | --- | --- |
|  | **Never drinker** | **Former drinker** | **Current drinker** | ***P*** |  | **Never drinker** | **Former drinker** | **Current drinker** | ***P*** |
| **Total** | 7527(46.85) | 1768(11.41) | 6454(41.74) | <0.001 |  | 23090(97.14) | 64(0.27) | 615(2.59) | <0.001 |
| **Age (years, mean ± SD)** | 58.61±12.16 | 60.86±9.81 | 53.21±12.21 | <0.001 |  | 55.03±12.13 | 53.50±14.59 | 51.36±11.93 |  |
| **Marital status** |  |  |  | <0.001 |  |  |  |  | 0.129 |
| Married/cohabiting | 6353(45.59) | 1596(11.45) | 5987(42.96) |  |  | 20683(97.07) | 60(0.28) | 564(2.65) |  |
| Unmarried/divorced/widowed | 904(58.17) | 172(11.07) | 478(30.76) |  |  | 2407(97.77) | 4(0.16) | 51(2.07) |  |
| **Education levels** |  |  |  | <0.001 |  |  |  |  | 0.071 |
| Primary school or illiteracy | 2830(54.11) | 672(12.85) | 1728(33.04) |  |  | 12081(97.37) | 35(0.28) | 289(2.34) |  |
| Junior high school | 3183(44.47) | 788(11.01) | 3187(44.52) |  |  | 8232(97.02) | 19(0.22) | 234(2.76) |  |
| High school or above | 1244(40.10) | 308(9.93) | 1550(49.97) |  |  | 2840(96.53) | 10(0.34) | 92(3.13) |  |
| **Income (RMB per month)** |  |  |  | <0.001 |  |  |  |  | <0.001 |
| <500 | 2940(52.47) | 725(12.94) | 1938(34.59) |  |  | 8196(97.44) | 28(0.33) | 187(2.22) |  |
| 500～ | 2237(45.39) | 559(11.34) | 2132(43.26) |  |  | 7786(97.58) | 17(0.21) | 176(2.21) |  |
| 1000～ | 2080(41.94) | 484(9.76) | 2395(48.30) |  |  | 7108(96.33) | 19(0.26) | 252(3.42) |  |
| **Smoking** |  |  |  | <0.001 |  |  |  |  | <0.001 |
| Never | 3213(65.58) | 212(4.33) | 1474(30.09) |  |  | 23022(97.22) | 61(0.26) | 598(2.53) |  |
| Former | 1167(36.83) | 826(26.07) | 1176(37.11) |  |  | 17(73.91) | 1(4.35) | 5(21.74) |  |
| Current | 2877(38.76) | 730(9.84) | 3815(51.40) |  |  | 51(78.46) | 2(3.08) | 12(18.46) |  |
| **Physical activity** |  |  |  | <0.001 |  |  |  |  | 0.088 |
| Low | 2649(47.93) | 737(13.33) | 2141(38.74) |  |  | 6997(97.34) | 17(0.24) | 174(2.42) |  |
| Moderate | 2100(48.80) | 481(11.18) | 1722(40.02) |  |  | 10215(97.27) | 32(0.30) | 255(2.43) |  |
| High | 2580(44.31) | 550(9.72) | 2602(45.97) |  |  | 5878(96.96) | 15(0.25) | 186(3.06) |  |
| **BMI** |  |  |  | <0.001 |  |  |  |  | 0.044 |
| Underweight | 269(64.35) | 46(11.00) | 103(24.64) |  |  | 518(97.00) | 5(0.94) | 11(2.06) |  |
| Normal | 3367(50.66) | 783(11.78) | 2496(37.56) |  |  | 8842(97.13) | 25(0.27) | 236(2.59) |  |
| Overweight | 2619(44.31) | 672(11.37) | 2620(44.32) |  |  | 9282(97.01) | 30(0.31) | 256(2.68) |  |
| Obesity | 972(39.58) | 255(10.38) | 1229(50.04) |  |  | 4381(97.46) | 4(0.09) | 110(2.45) |  |
| **SBP (mmHg, mean ± SD)** | 126.07±18.82 | 127.69±19.41 | 127.16±18.04 | <0.001 |  | 125.64±20.89 | 122.55±19.79 | 119.03±18.35 | <0.001 |
| **DBP (mmHg, mean ± SD)** | 77.36±11.57 | 78.21±11.87 | 80.61±12.08 | <0.001 |  | 77.02±11.40 | 74.31±10.86 | 75.23±11.45 | <0.001 |
| **TC (mmol/L, mean ± SD)** | 4.57±0.94 | 4.56±0.97 | 4.75±0.97 | <0.001 |  | 4.84±1.00 | 4.71±0.95 | 4.63±0.94 | <0.001 |
| **FPG (mmol/L, mean ± SD)** | 5.50±1.50 | 5.64±1.63 | 5.55±1.48 | 0.003 |  | 5.56±1.52 | 5.72±1.60 | 5.30±1.29 | <0.001 |
| **T2DM (yes)** | 667(47.27) | 231(16.37) | 513(36.36) | <0.001 |  | 2257(98.26) | 8(0.35) | 32(1.39) | 0.001 |

Abbreviation: SD, standard deviation; RMB, Renminbi; BMI, body mass index; SBP, systolic blood pressure; DBP, diastolic blood pressure; TC, total cholesterol; FPG, fasting plasma glucose. Income (RMB per month), Per capita monthly income; T2DM: type 2 diabetes mellitus.

**Supplementary Table 2. Characteristics of the participants by WHO risk drinking levels by sex.**

| **WHO risk drinking levels** | **Total** |  | **Men** |  | **Women** |
| --- | --- | --- | --- | --- | --- |
| **Never** | 30432(77.52) |  | 7326(47.30) |  | 23106(97.21) |
| **Low-risk** | 5447(13.87) |  | 4909(31.69) |  | 538(2.26) |
| **Medium-risk** | 1848(4.71) |  | 1763(11.38) |  | 85(0.36) |
| **High-risk** | 1532(3.90) |  | 1492(9.63) |  | 40(0.17) |

low-risk drinkers (>0 to 25 g/d for men,>0 to 15 g/d for women), medium-risk drinkers (>25 to 60 g/d for men, >15 to 40 g/d for women), high-risk drinkers (>60 g/d for men, >40 g/d for women).

**Supplementary Table 3. Summary of participants****’ age of starting to drink, duration of drinking and alcohol intake by sex.**

| **Drinking** | Mean | Min |  | Quantiles | | |  | Max | IQR |
| --- | --- | --- | --- | --- | --- | --- | --- | --- | --- |
|  |  |  |  | Q25 | Q50 | Q75 |  |  |  |
| **Total** |  |  |  |  |  |  |  |  |  |
| Age of starting to drink | 23.19 | 7 |  | 18 | 20 | 25 |  | 77 | 7 |
| Duration of drinking | 30.77 | 1 |  | 22 | 31 | 40 |  | 70 | 18 |
| Alcohol intake (g/d) |  |  |  |  |  |  |  |  |  |
| **Men** |  |  |  |  |  |  |  |  |  |
| Age of starting to drink | 22.47 | 7 |  | 18 | 20 | 25 |  | 72 | 7 |
| Duration of drinking | 31.48 | 1 |  | 23 | 31 | 40 |  | 70 | 17 |
| Alcohol intake (g/d) | 19.94 | <0.001 |  | <0.001 | 1.15 | 19.72 |  | 532.6 | 19.72 |
| **Women** |  |  |  |  |  |  |  |  |  |
| Age of starting to drink | 33.47 | 7 |  | 21 | 30 | 43 |  | 77 | 22 |
| Duration of drinking | 23.37 | 1 |  | 8 | 20 | 35 |  | 69 | 27 |
| Alcohol intake (g/d) | 0.29 | <0.001 |  | <0.001 | <0.001 | <0.001 |  | 246.57 | <0.001 |

IQR, interquartile range.

**Supplementary Table 4. The distribution of age of starting to drink by sex according age.**

| **Age** |  | **Age of starting to drink (n(%))** | | |  | **Age of initiation of sobriety (n(%))** | | |
| --- | --- | --- | --- | --- | --- | --- | --- | --- |
|  |  | **Total** | **Men** | **Women** |  | **Total** | **Men** | **Women** |
| **<18** |  | 2206(25.96) | 2142(26.99) | 64(11.41) |  | 1(0.05) | 0.00 | 1(1.61) |
| **18~29** |  | 4826(56.79) | 4640(58.46) | 186(33.16) |  | 55(3.02) | 47(2.67) | 8(12.90) |
| **30~39** |  | 925(10.88) | 793(9.99) | 132(23.53) |  | 169(9.29) | 162(9.22) | 7(11.29) |
| **40~49** |  | 299(3.52) | 213(2.68) | 86(15.33) |  | 413(22.69) | 396(22.53) | 17(27.42) |
| **50~59** |  | 146(1.72) | 95(1.2) | 51(9.09) |  | 601(33.02) | 585(33.28) | 16(25.81) |
| **60~69** |  | 81(0.95) | 48(0.6) | 33(5.88) |  | 482(26.48) | 472(26.85) | 10(16.13) |
| **70~79** |  | 15(0.18) | 6(0.08) | 9(1.6) |  | 99(5.44) | 96(5.46) | 3(4.84) |

**Supplementary Table 5. The association of the type of alcoholic with T2DM by sex.**

| **Type of alcoholic** |  | **Model 1** |  | **Model 2** |  | **Model 3** |
| --- | --- | --- | --- | --- | --- | --- |
|  |  | **OR (95%CI)** |  | **OR (95%CI)** |  | **OR (95%CI)** |
| **Total** |  |  |  |  |  |  |
| Liquor |  | 1.002(1.001, 1.003) |  | 1.002(1.001, 1.003) |  | 1.002(1.000, 1.003) |
| Beer |  | 0.996(0.987, 1.006) |  | 1.000(0.990, 1.009) |  | 1.000(0.990, 1.009) |
| Red wine |  | 0.996(0.940, 1.055) |  | 0.993(0.934, 1.055) |  | 0.998(0.941, 1.058) |
| Other |  | 1.018(0.958, 1.082) |  | 1.022(0.960, 1.088) |  | 1.030(0.962, 1.102) |
| **Men** |  |  |  |  |  |  |
| Liquor |  | 1.002(1.001, 1.003) |  | 1.002(1.001, 1.003) |  | 1.002(1.000, 1.003) |
| Beer |  | 0.996(0.986, 1.006) |  | 1.000(0.991, 1.010) |  | 1.000(0.991, 1.010) |
| Red wine |  | 0.987(0.919, 1.060) |  | 0.983(0.910, 1.063) |  | 0.994(0.921, 1.072) |
| Other |  | 1.022(0.966, 1.082) |  | 1.025(0.964, 1.091) |  | 1.036(0.969, 1.108) |
| **Women** |  |  |  |  |  |  |
| Liquor |  | 0.999(0.985, 1.014) |  | 0.996(0.980, 1.012) |  | 0.998(0.983, 1.014) |
| Beer |  | 0.949(0.844, 1.066) |  | 0.983(0.830, 1.060) |  | 0.937(0.828, 1.062) |
| Red wine |  | 1.034(0.936, 1.142) |  | 1.036(0.913, 1.176) |  | 0.994(0.790, 1.250) |
| Other |  | 0.186(0.000, 225.981) |  | *NA* |  | *NA* |

OR: odds ratios, CI: confidence intervals.

Model 1: unadjusted; Model 2: adjusted for age, gender, education level, marital status, and smoking; Model 3: adjusted for model 2 plus family histories of T2DM, more vegetables and fruits intake, high fat diet, physical activity.


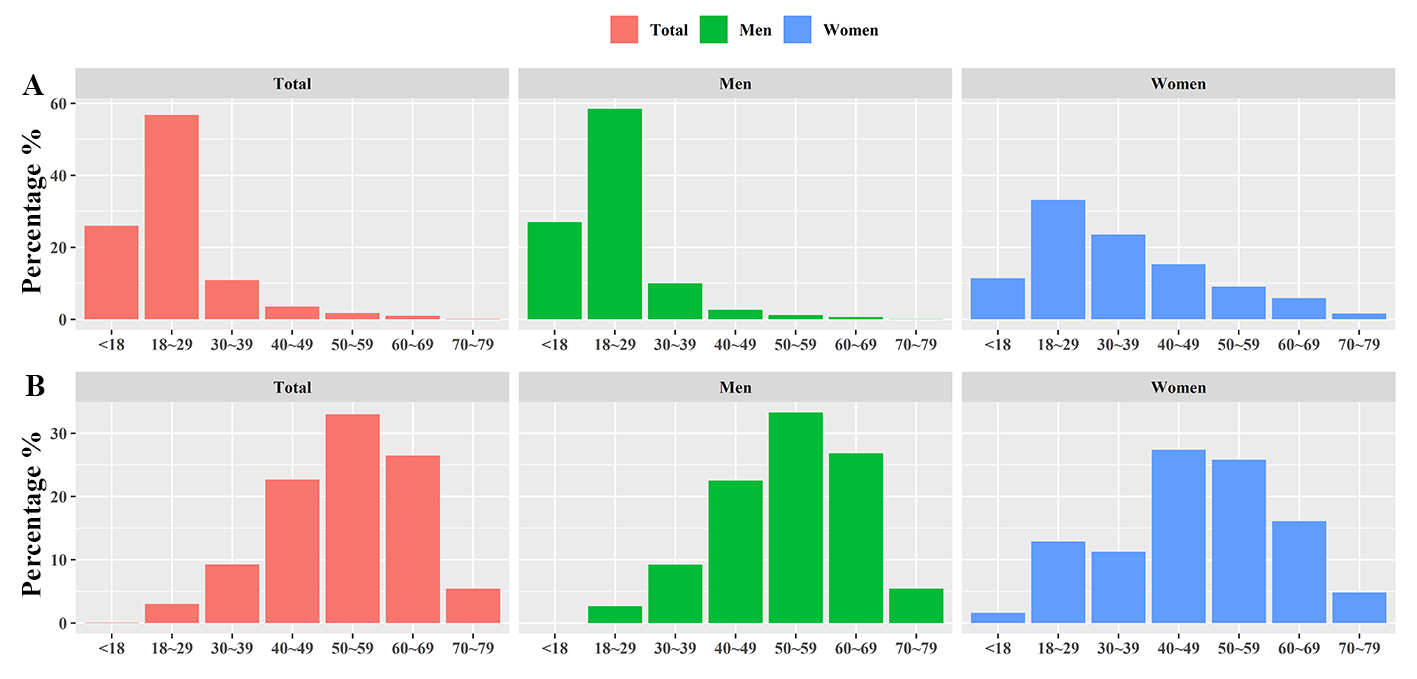


**Supplementary figure 1.** The distribution of age of starting to drink and initiation of sobriety by sex according age. A: the distribution of age of starting to drink; B: the distribution of age of initiation of sobriety.


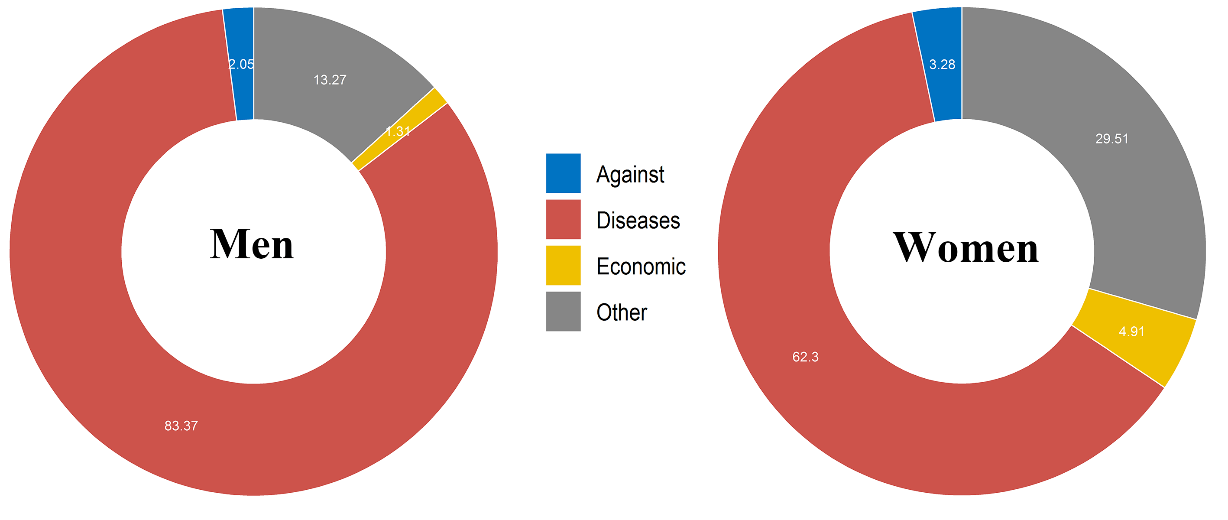


**Supplementary figure 2.** The distribution of reasons for quitting alcohol in different sexes.


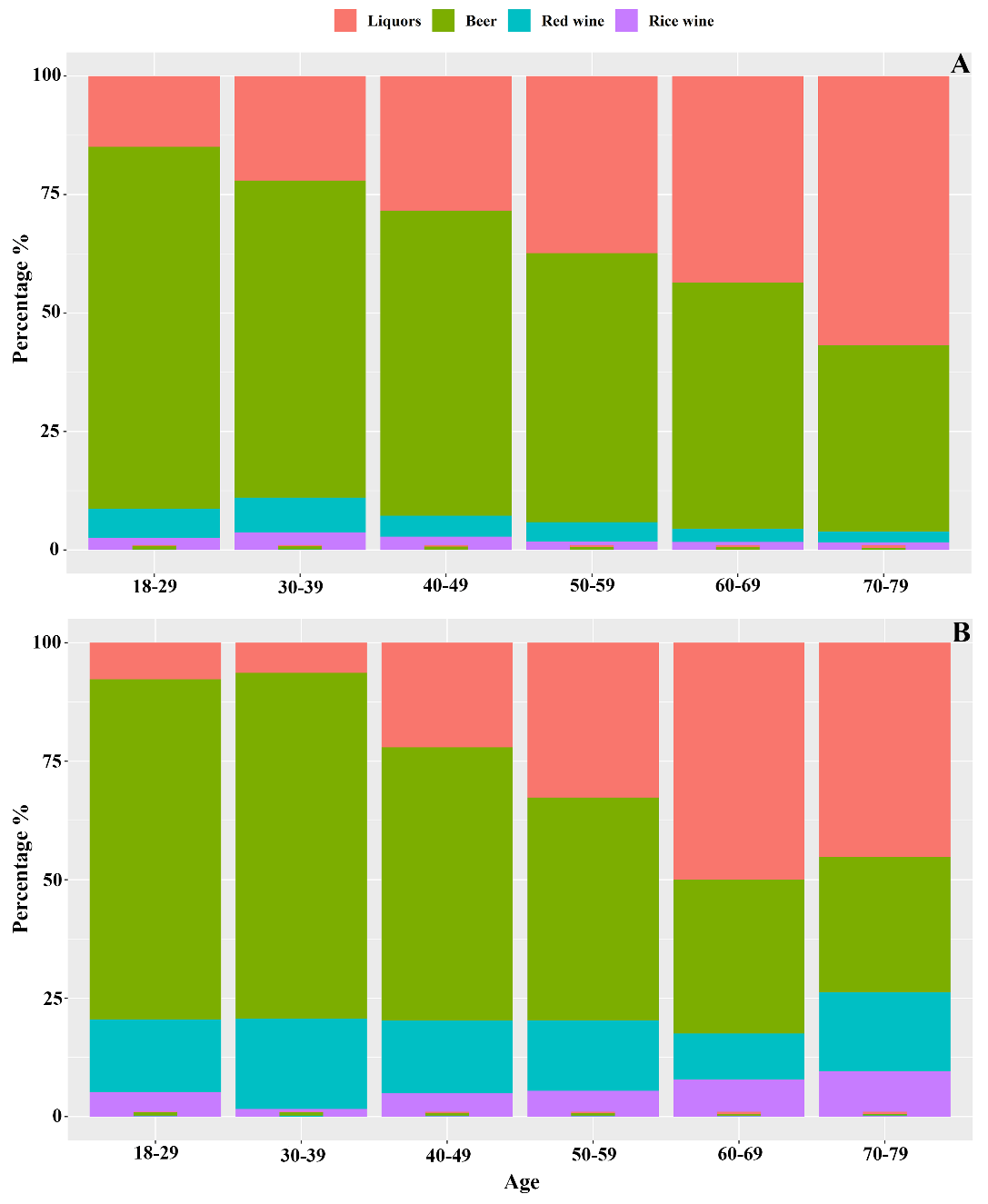


**Supplementary figure 3.** The type of alcoholic beverage preferred according age by sexes. A: men; B: women.


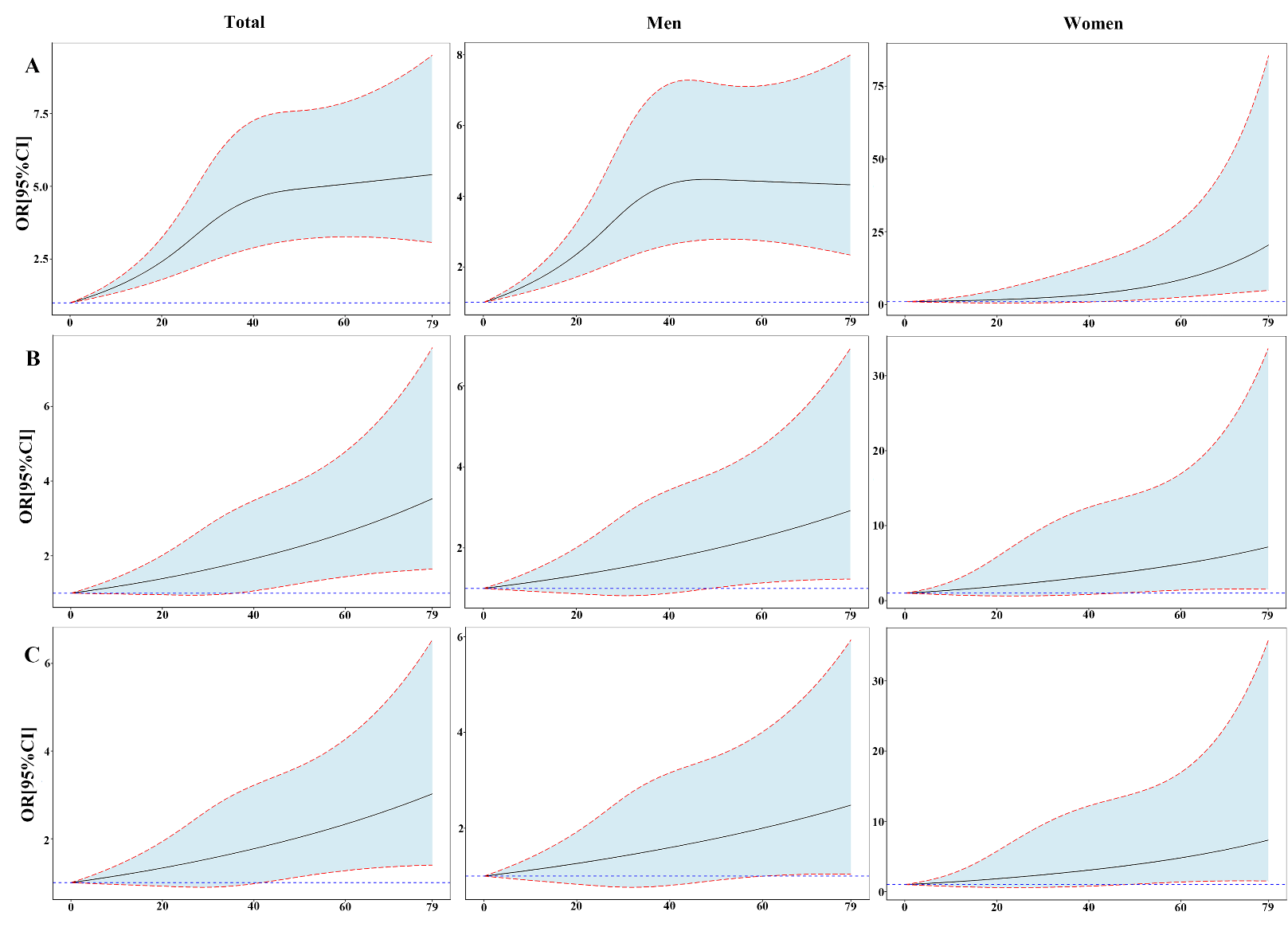


**Supplementary figure 4.** Mean and 95% confidence intervals of T2DM along with the changes of number of years of consuming alcohol from restricted cubic splines by gender. A, Model 1: unadjusted; B, Model 2: adjusted for age, gender, education level, marital status, and smoking; C, Model 3: adjusted for model 2 plus family histories of T2DM, more vegetables and fruits intake, high fat diet, physical activity.


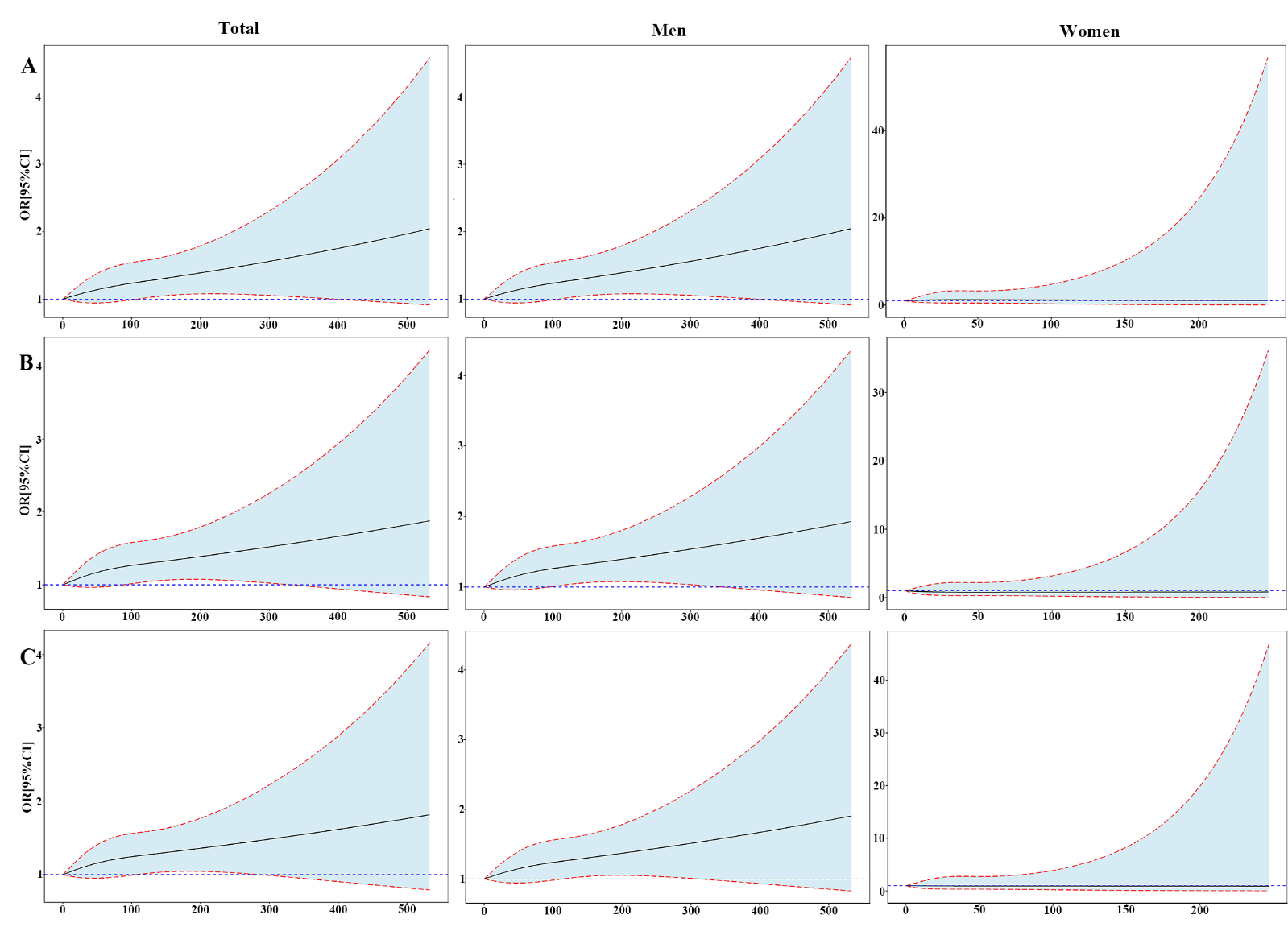


**Supplementary figure 5.** Mean and 95% confidence intervals of T2DM along with the changes of alcohol intake from restricted cubic splines by gender. A, Model 1: unadjusted; B, Model 2: adjusted for age, gender, education level, marital status, and smoking; C, Model 3: adjusted for model 2 plus family histories of T2DM, more vegetables and fruits intake, high fat diet, physical activity.


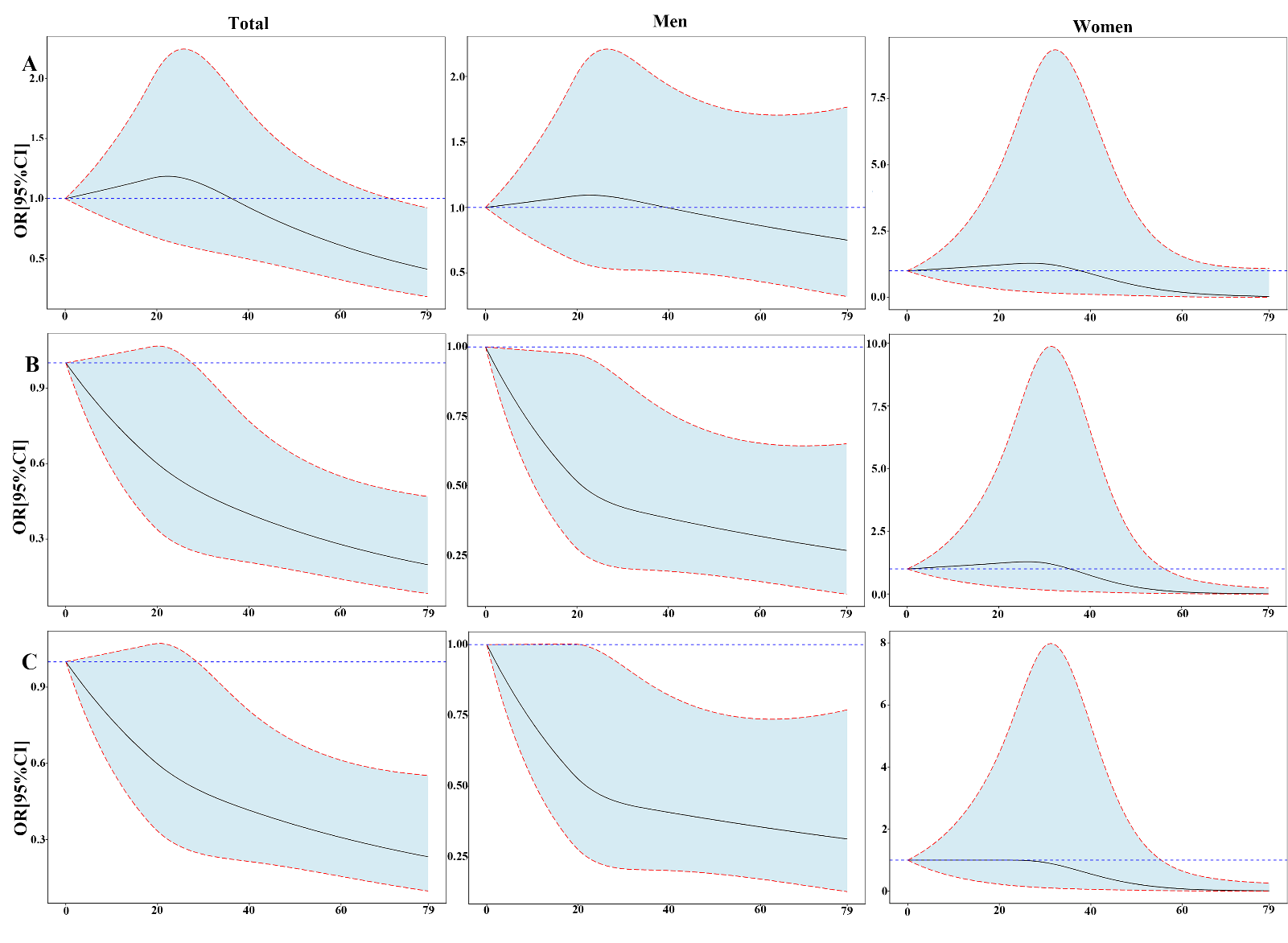


**Supplementary figure 6.** Mean and 95% confidence intervals of T2DM along with the changes of age of starting to consume alcohol from restricted cubic splines by gender. A, Model 1: unadjusted; B, Model 2: adjusted for age, gender, education level, marital status, and smoking; C, Model 3: adjusted for model 2 plus family histories of T2DM, more vegetables and fruits intake, high fat diet, physical activity.
